# Prevalence and factors associated with non-vaccination: a vaccination coverage survey among children born in Ceará, Brazil, 2017-2018

**DOI:** 10.1590/0037-8682-0270-2025

**Published:** 2026-04-10

**Authors:** Adjoane Maurício Silva Maciel, Carla Magda Allan Santos Domingues, Anderson Fuentes Ferreira, Ana Paula França, Nádia Maria Girão Saraiva de Almeida, Maria Vaudelice Mota, José Cássio de Moraes, Alberto Novaes Ramos

**Affiliations:** 1 Universidade Federal do Ceará, Faculdade de Medicina, Programa de Pós-Graduação em Saúde Pública, Fortaleza, CE, Brasil.; 2 Secretaria da Saúde do Estado do Ceará, Fortaleza, CE, Brasil.; 3 Organização Pan-Americana da Saúde, Brasília, DF, Brasil.; 4 Faculdade de Ciências Médicas da Santa Casa de São Paulo, São Paulo, SP, Brasil.; 5 Universidade Federal do Ceará, Fortaleza, CE, Brasil.; 6 Universidade Federal do Ceará, Faculdade de Medicina, Departamento de Saúde Comunitária, Fortaleza, CE, Brasil.

**Keywords:** Vaccine-preventable diseases, Disease susceptibility, Immunization programs, Health surveys

## Abstract

**Background:**

The current prevalence and factors associated with non-vaccination in children born between 2017 and 2018, considering vaccination status up to 24 months of age in the State of Ceará, should be assessed to justify the need to identify the population eligible for timely vaccination with valid doses to ensure optimal protection against vaccine-preventable diseases. This study aimed to estimate the prevalence and analyze the sociodemographic factors associated with non-vaccination among children born between 2017 and 2018 residing in Fortaleza and Sobral, Ceará State, Northeast Brazil.

**Methods:**

A population-based household survey of the 2017-2018 birth cohort was conducted using cluster sampling in Fortaleza and Sobral. The prevalence of non-vaccination, including no-dose and incomplete-dose categories, was estimated using prevalence ratios from Poisson regression with robust variance and survey design adjustment.

**Results:**

Of the children, 54.55% were not fully vaccinated, and 9.91% did not receive a single valid dose of any vaccine (Fortaleza: 10.46%; Sobral: 2.71%). Missing doses were mainly for meningitis (29.14%), rotavirus diarrhea (25.46%), and pneumonia and meningitis (24.44%). Non-vaccination was associated with maternal ethnicity, specifically White (Caucasian) (aPR, 2.07; 95% CI, 1.08-3.94; p = 0.028), and with the use of private vaccination services (aPR, 1.72; 95% CI, 1.46-2.04; p < 0.001).

**Conclusions:**

Non-vaccination was highly prevalent among children born between 2017 and 2018 in Ceará. The findings highlight the need to strengthen and reorient vaccination efforts across sectors and to implement catch-up strategies for children who missed valid doses.

## INTRODUCTION

The National Immunization Program (In Portuguese: Programa Nacional de Imunizações [PNI]), established in Brazil in 1973, has led to a change in the epidemiological scenario of vaccine-preventable diseases in the country, legitimizing vaccination as a relevant and safe public health intervention^1^ ^,^ ^2^. The direct costs of hospitalizations related to vaccine-preventable diseases in the Unified Health System (In Portuguese: Sistema Único de Saúde [SUS]) between 2008 and 2018 indicate that proven-effective public health interventions, such as vaccination, have indirectly reduced health-sector spending by more than R$ 389 million^3^ ^,^ ^4^.

However, factors potentially associated with increased susceptibility to diseases due to non-vaccination include a lack of clear awareness of the seriousness of the current vaccination coverage scenario, a sense of false security, the need to keep the vaccination schedule up-to-date, financial and geographical access barriers, infrastructure problems, socioeconomic and cultural issues, public policies and governance, and the availability of production chains for immunobiological research and innovation centers in the country^1^ ^,^ ^2^.

Between 2013 and 2015, the measles virus was reintroduced into Brazil, with cases occurring in the states of Bahia, Paraíba, and Pernambuco, and an epidemic in the State of Ceará^5^. In this context, a vaccination survey of a cohort of live births between 2017 and 2018 confirmed low vaccination coverage, below the PNI parameters, for most immunobiologicals recommended in the childhood vaccination schedule. Two important municipalities in this state, Fortaleza and Sobral, show greater susceptibility to vaccine-preventable diseases^6^.

The World Health Organization (WHO) proposed an agenda to strengthen immunization programs worldwide, known as Immunization Agenda 2030^7^ ^,^ ^8^. The proposed actions include improving policies and operational plans, monitoring vaccination data, ensuring access to immunobiologicals, and preventing vaccine-preventable diseases^8^. Thus, the current prevalence and factors associated with non-vaccination in children up to 24 months of age in the State of Ceará must be assessed to justify the need to identify the population eligible for timely vaccination with valid doses to ensure optimal protection against vaccine-preventable diseases. Given these challenges, this study aimed to estimate the prevalence and analyze the sociodemographic factors associated with non-vaccination in children born in 2017-2018, considering the vaccination status up to 24 months (valid doses), in the municipalities of Fortaleza (capital) and Sobral in the State of Ceará, Northeast Brazil.

## METHODS

### Design

We conducted a household-based population survey of the 2017-2018 birth cohort assessing vaccination status up to 24 months of age (valid doses) in two municipalities in the State of Ceará, within the scope of the *“Vaccination coverage survey in the capitals of 26 states, in the Federal District and in 12 inland municipalities in children born in 2017*-*2018 living in urban areas”* ^9^ ^-^ ^11^.

### Study location and period

The municipality of Fortaleza (capital) has an estimated resident population (2020) of 2,686,612, of which 3.7% (99,281) are children aged between 0 and 2 years, and 58.0% are covered by Primary Healthcare (PHC). The municipality of Sobral, located in the interior of the state, has 210,711 inhabitants, of which 4.3% (9,102) are children aged 0-2 years. In Sobral, the population is fully covered by PHC. In the two municipalities, vaccination services included public facilities (Fortaleza, n=166; Sobral, n=45) and private facilities (Fortaleza, n=37; Sobral, n=4)^12^. The study was conducted between September 2020 and March 2022. 

### Population and sample

The study population consisted of live births between 2017 and 2018 in the municipalities of Fortaleza and Sobral in the State of Ceará^13^. The sampling procedure consisted of two stages: census tracts were selected, followed by the selection of eligible households with at least one live birth between 2017 and 2018.

Urban census tracts were ecologically defined in socioeconomic strata and classified spatially at four levels (A, B, C, and D), characterized by the lowest (D) represented by regions with the worst income and schooling indicators, to the highest socioeconomic level (A), with a proportion of literate heads of households and heads of households with an income of ≥20 minimum wages^12^.

Identified urban census tracts were grouped according to geographical proximity, composed of equal numbers of children registered in the Live Birth Information System (In Portuguese: Sistema de Informações de Nascidos Vivos [Sinasc]). The tracts were georeferenced using child and parentage data, as well as addresses from the Sinasc nominal database. The sample size was calculated according to the *“National Vaccination Coverage Survey 2020: methods and operational aspects”.*

In each municipality surveyed, one to four sample groups were assigned based on the size of the live-birth population in each municipality^9^. Based on these data and to facilitate comparisons between socioeconomic strata, the sample included conglomerates of 452 children per group, with 113 children from each of the four socioeconomic strata. The total number of children was 1,808 in Fortaleza (four sample groups) and 452 in Sobral (one sample group).

### Data collection

Teams of interviewers collected household data from the parents/guardians of children born alive between 2017 and 2018 with a Sinasc address and an active search in the conglomerates using an electronic questionnaire consisting of open and closed questions to characterize the child, and a photographic record of the child's vaccination card for data evaluation by immunization experts. For individuals without vaccination cards, information was collected using the National Immunization Program Information System (SI-PNI). 

### Variables

Sociodemographic variables were characterized according to socioeconomic strata (A, B, C, and D) and municipality of residence (Fortaleza and Sobral). Families were assessed according to the following variables: agglomeration, assessed as having four or more persons per room (yes or no), Bolsa Família Program (BFP) beneficiary families (yes or no), grandparents living in the household (yes or no), and monthly family income (BRL) (≤1,000.00, 1,001.00-3,000.00, 3,001.00-8,000.00, or ≥8,001.00). Regarding the mothers, we assessed their schooling in terms of years of study (0-8, 9-12, 13-15, or ≥16); age group at childbirth (years) (<20, 20-34, or ≥35 years); ethnicity (Caucasian, Afro-Brazilian/Afro-descendant, Mixed/Pardo Brazilians, Asian-descendant, or Indigenous); paid work (yes or no); marital status (with or without a partner); number of live children per mother (1 child or ≥2 children); and type of delivery (vaginal or caesarean).

For children, the following variables were evaluated: vaccination card (yes or no), use of private vaccination services (yes or no), sex (male or female), birth order (first, second, third, fourth, or higher), ethnicity (White, Black, Brown, Asian-descendant, or Indigenous), and daycare/school attendance (yes or no). 

For the outcome variables, the following concepts were considered:

*Non-vaccination*: Children born between 2017 and 2018 were classified as not vaccinated according to the schedule up to 24 months of age when they had not received the valid doses^9^ recommended by the Brazilian National Immunization Program (PNI)^14^ schedule. Valid doses were defined based on age eligibility and minimum recommended intervals, where applicable. Two categories were considered: no vaccination/no valid doses, corresponding to children who had not received any vaccine or any valid dose of any vaccine; and *incomplete vaccination/incomplete valid doses*, corresponding to children who had not received at least one, two, or three valid doses in schedules comprising two, three, or four doses, respectively.

***Complete vaccination/complete doses*** referred to the number and proportion of children born between 2017 and 2018 who received complete valid doses scheduled up to 24 months^9^ proposed by the PNI.

Yellow fever was not included in the analysis of the vaccination schedule, as it was not recommended on routine services in the State of Ceará, including the capital Fortaleza, during the research period; however, it was presented individually considering the context of the municipalities of Sobral and Cariri (not included in the present municipal analysis), where the process of implementation of the vaccine began in 2020.

The vaccine-preventable diseases included in the non-vaccination analysis, either individually or in groups, were those prevented by the vaccines provided by the PNI, considering the national vaccination calendar and the doses planned and not applied, as well as the targets for vaccination coverage (Table 1).

**TABLE 1:** Recommended vaccines and vaccination coverage targets for children up to 24 months of age, based on the 2023 Ceará State vaccination calendar*.

Age	Vaccine	Dose	Target
**At birth**	BCG	Single	90%
	Hepatitis B	1^st^	95%
**2 months**	Pentavalent	1^st^	95%
	Inactivated Poliovirus Vaccine (IPV)	1^st^	95%
	10-valent Pneumococcal Conjugate Vaccine (PCV10)	1^st^	95%
	Rotavirus Vaccine	1^st^	90%
**3 months**	Meningococcal C Vaccine	1^st^	95%
**4 months**	Pentavalent	2^nd^	95%
	Inactivated Poliovirus Vaccine	2^nd^	95%
	10-valent Pneumococcal Conjugate Vaccine (PCV10)	2^nd^	95%
	Rotavirus Vaccine	2^nd^	90%
**5 months**	Meningococcal C Vaccine	2^nd^	95%
**6 months**	Pentavalent	3^rd^	95%
	Inactivated Poliovirus Vaccine	3^rd^	95%
**12 months**	MMR	1^st^	95%
	Meningococcal C Vaccine	Booster	95%
	10-valent Pneumococcal Conjugate Vaccine (PCV10)	Booster	95%
**15 months**	DTP	1^st^ booster	95%
	Bivalent Oral Polio Vaccine (bOPV)	1^st^ booster	95%
	Hepatitis A	Single	95%
	MMR	2^nd^	95%
	Varicella Vaccine	1^st^	95%

*Adapted from the 2023 vaccination calendar of the Ceará State Health Department. For 24-month vaccination coverage, the vaccination schedules proposed for 12 and 15 months were used. **BCG:** Bacille Calmette-Guérin; Pentavalent: diphtheria, tetanus, pertussis, and *Haemophilus influenzae b;* **IPV:** Inactivated Poliovirus Vaccine; **MMR:** measles, rubella, and mumps; **DTP:** diphtheria, tetanus, and pertussis.

The dependent variable was non-vaccination status (*non*-*vaccination/no doses + incomplete vaccination/incomplete doses*), with vaccination status (*complete vaccination/complete dose*) used as the reference group.

The results are presented based on whether the child was unvaccinated (received no valid doses), incompletely vaccinated (received incomplete valid doses), or fully vaccinated (received complete valid doses) according to the vaccination schedule recommended in the PNI calendar.

The date of birth and administration of each vaccine during the first 24 months of life were obtained to confirm the age of the child at the time of vaccination.

### Analysis

Sample weights were calculated for each household/child based on the probability of selection and calibrated to population groups with adjustments for non-response and design effect^9^ ^,^ ^10^.

Weighted estimates were calculated for the variables included in the study and for the vaccine-preventable diseases analyzed, with corresponding 95% confidence intervals (CI). Significance was set at p < 0.05^9^ ^,^ ^10^.

The simple and relative proportions of the study variables were calculated and presented in tables and graphs.

The association between explanatory variables and the outcome of non-vaccination was evaluated using univariate analyses and prevalence calculations, with 95% CI and prevalence ratios (PRs) estimated using Poisson regression with robust variance^15^. Analyses were conducted at the individual level, and the outcomes were binary. This approach is widely used for cross-sectional analyses with common outcomes in which PRs are preferred over odds ratios for interpretability. All models incorporated a complex sampling design to obtain appropriate variance estimates. The analyses were conducted in two stages: first, crude analyses were performed; then, multivariable analyses were conducted to adjust for other variables of interest and sample weights.

Variables with p ≤ 0.05 were considered statistically significant in the final model; for categorical variables, p-values correspond to category-specific comparisons with the reference group (survey-weighted Wald tests). Crude and adjusted PRs and 95% CIs were estimated using survey-weighted procedures (Stata svy), with design-based variance estimation (Taylor series linearization) to account for sampling weights and complex sample structure^16^.

Collinearity among variables was assessed using the variance inflation factor (VIF). VIF values are ≥1 and have no upper bound; values >5 indicate problematic multicollinearity. 

Stata® 15 software (StataCorp LLC, College Station, Texas, TX, United States, Stata Statistical Software: Release 15. College Station, TX, StataCorp LLC) was used for the analysis using the svy framework to analyze survey data with a complex sample^11^.

### Ethical aspects

This study was approved by the Human Research Ethics Committee of the Institute of Collective Health at the Federal University of Bahia (UFBA no. 3.366.818) and Irmandade da Santa Casa de São Paulo (no. 4.380.019). Written informed consent was obtained from the parents/guardians before the interview and review of the vaccination cards.

## RESULTS

The final study sample comprised 2,077 children living in the municipalities of Fortaleza and Sobral in Ceará.

The majority of children who were not vaccinated (no doses or incomplete doses) were female (58.18%), the second child in the birth order (62.23%), of White ethnicity (64.18%), attending daycare or school (57.46%), of a higher socioeconomic stratum (A) (76.24%), had used a private vaccination service at least once (85.75%), had a monthly family income ≥ 8,001.00 (95.38%), and had no access to the BFP (57.76%) (Table 2). 

The maternal profile showed that most mothers had ≥16 years of schooling (76.76%), were aged ≥20 years (65.30%), were White (69.58%), and had a partner (55.48%) (Table 2).

**TABLE 2:** Sociodemographic and family characteristics of children up to 24 months of age in cohorts of live births in 2017 and 2018, according to vaccination status and 95% CI, living in the municipalities of Fortaleza and Sobral, Northeast Brazil (N=2,077).

Sociodemographic characteristics	Non-vaccination			Complete vaccination
	Non-vaccination	Incomplete vaccination	Non-vaccination + incomplete vaccination	
	% (95%CI)	% (95%CI)	% (95%CI)	% (95%CI)
**Total**	**9.91 (6.42-15.00)**	**44.64 (40.46-48.91)**	**54.55 (48.67-60.31)**	**45.45 (39.69-51.33)**
Socio-economic strata				
A	19.50 (7.56-41.77)	56.74 (49.20-63.99)	76.24 (55.45-89.21)	23.76 (10.79-44.55)
B	7.18 (3.83-13.08)	43.88 (38.54-49.37)	51.06 (43.78-58.30)	48.94 (41.70-56.22)
C	8.26 (4.96-13.43)	39.24 (30.03-49.28)	47.50 (38.62-56.53)	52.50 (43.47-61.38)
D	7.11 (4.53-10.97)	43.85 (39.44-48.36)	50.95 (45.70-56.19)	49.05 (43.81-54.30)
**Municipalities of residence (Ceará-CE)**				
Fortaleza (capital)	10.46 (6.75-15.88)	44.22 (39.78-48.74)	54.68 (48.40-60.81)	45.32 (39.19-51.60)
Sobral (inland)	2.71 (0.58-11.74)	50.19 (40.50-59.86)	52.90 (42.98-62.59)	47.10 (37.41-57.02)
**Use of private vaccination service**				
Yes	21.18 (8.84-42.71)	64.56 (48.93-77.60)	85.75 (69.91-93.97)	14.25 (6.03-30.09)
No	7.58 (5.51-10.34)	41.13 (36.30-46.13)	48.71 (43.63-53.81)	51.29 (46.19-56.37)
**Monthly family income (BRL)**				
≤1,000.00	5.75 (3.64-8.97)	47.87 (41.52-54.28)	53.62 (47.11-60.01)	46.38 (39.99-52.89)
1,001.00−3,000.00	10.38 (6.93-15.27)	39.44 (34.63-44.48)	49.82 (44.06-55.59)	50.18 (44.41-55.94)
3,001.00−8,000.00	6.11 (1.49-21.85)	28.71 (15.80-46.37)	34.82 (19.36-54.32)	65.18 (45.68-80.64)
≥8,001.00	12.44 (3.58-35.21)	82.94 (60.95-93.81)	95.38 (86.53-98.52)	4.62 (1.48-13.47)
Don't know/didn't answer	29.92 (13.00-54.95)	44.79 (37.32-52.50)	74.71 (40.22-92.84)	25.29 (7.16-59.78)
**Maternal characteristics**				
**Ethnicity**				
Caucasian	16.56 (7.46-32.81)	53.03 (41.96-63.80)	69.58 (55.41-80.81)	30.42 (19.19-44.59)
Afro-Brazilian / Afro-descendant	3.05 (0.55-15.27)	25.44 (12.69-44.47)	28.49 (14.22-48.92)	71.51 (51.08-85.78)
Mixed/ Pardo Brazilians	7.90 (5.45-11.32)	44.12 (39.69-48.65)	52.02 (46.94-57.06)	47.98 (42.94-53.06)
Asian-descendant	0.00 (0.00-0.00)	18.56 (6.09-44.45)	18.56 (6.09-44.45)	81.44 (55.55-93.91)
Indigenous (Amerindians)	0.00 (0.00-0.00)	12.22 (0.85-69.38)	12.22 (0.85-69.38)	87.78 (30.62-99.15)
Don't know/didn't answer	16.91 (6.25-38.31)	37.82 (15.53-66.80)	54.73 (31.13-76.38)	45.27 (23.62-68.87)
**Number of live children per mother**				
1	6.20 (3.77-10.02)	46.85 (40.19-53.62)	53.05 (45.58-60.39)	46.95 (39.61-54.42)
2 or more	11.98 (7.09-19.52)	43.41 (38.77-48.17)	55.39 (47.64-62.88)	44.61 (37.12-52.36)
**Characteristics of the child**				
**Ethnicity**				
White	14.48 (7.44-26.31)	49.70 (39.61-59.81)	64.18 (51.39-75.23)	35.82 (24.77-48.61)
Black	23.07 (6.83-55.09)	30.29 (16.51-48.86)	53.37 (33.67-72.07)	46.63 (27.93-66.33)
Brown	6.22 (4.24-9.04)	42.25 (37.06-47.62)	48.47 (42.56-54.43)	51.53 (45.57-57.44)
Asian-descendant	10.95 (1.33-52.87)	39.40 (8.60-81.79)	50.35 (14.33-86.01)	49.65 (13.99-85.67)
Indigenous	0.00 (0.00-0.00)	0.00 (0.00-0.00)	0.00 (0.00-0.00)	100.00 (100.00-100.00)
**Attendance at daycare/school**				
Yes	9.14 (3.77-20.52)	48.32 (43.13-53.55)	57.46 (47.82-66.57)	42.54 (33.43-52.18)
No	10.65 (7.63-14.69)	41.10 (34.93-47.56)	51.76 (45.71-57.75)	48.24 (42.25-54.29)

**CI:** confidence interval. Estimates of 95%CI are derived from the sampling design.

**FIGURE 1:**
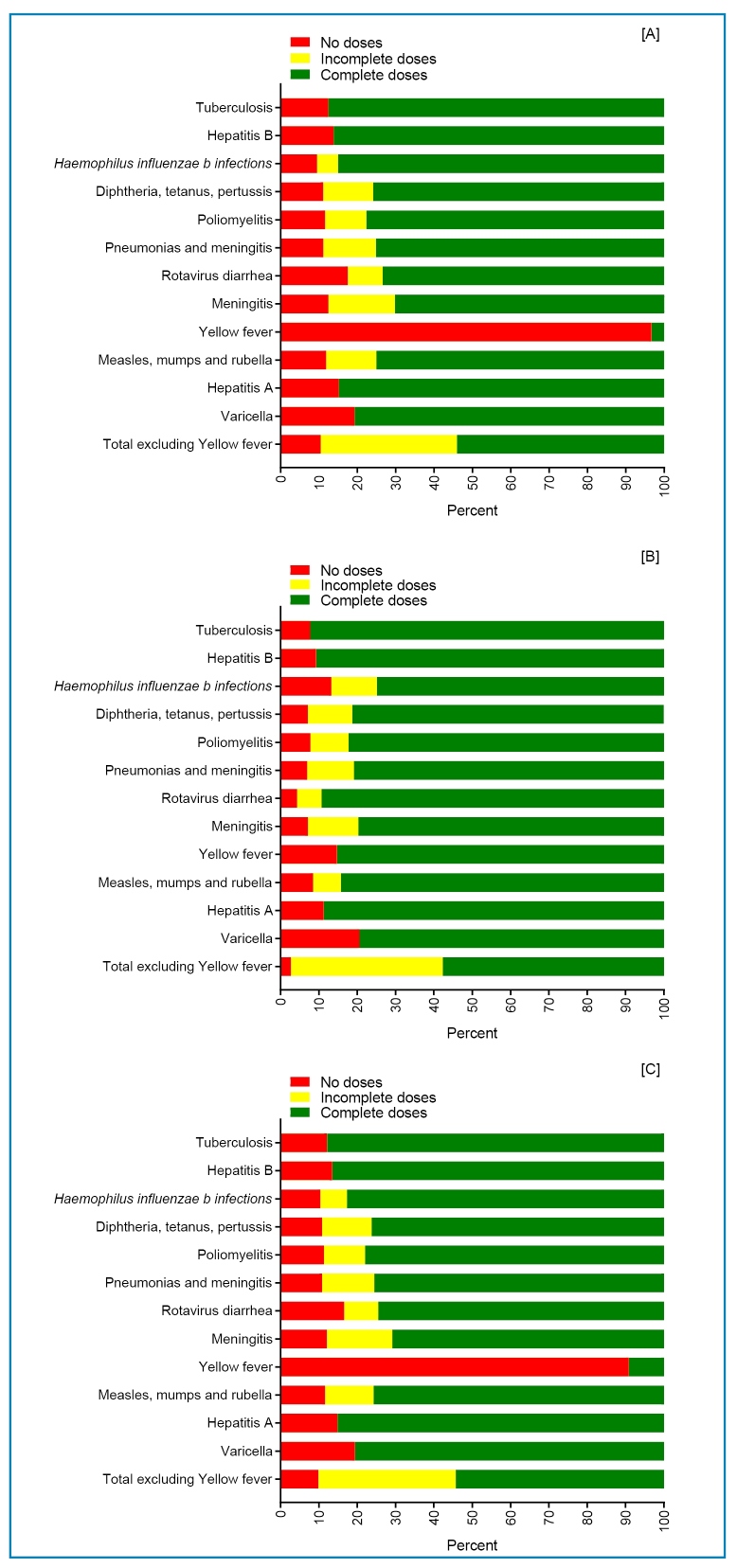
Non-vaccination against vaccine-preventable diseases among children up to 24 months of age in live birth cohorts in 2017 and 2018, according to valid doses, residing in the municipalities of Fortaleza **[A]** and Sobral **[B]**, and in the State of Ceará **[C]**, Northeast Brazil (N=2,077).

Among all children surveyed in the two municipalities, the non-vaccination prevalence was 54.55%. Stratified by municipality, the prevalence rates were 54.68% and 52.90% in Fortaleza and Sobral, respectively. The prevalence of non-vaccination was 54.68% in Fortaleza and 52.90% in Sobral, including children without any dose (9.91%) (Fortaleza: 10.46%; Sobral: 2.71%) (Figure 1A and Figure 1B, respectively).

A higher proportion of children with incomplete doses was noted for meningitis (17.04%) and pneumonia (13.59%) (Figure 1C).

The presence of children without any dose was found in higher proportions for the following diseases: varicella (19.41%), rotavirus diarrhea (16.59%), and hepatitis A (14.90%) (Figure 1C), with occurrences recorded from birth; hepatitis B (13.48%); and Bacillus Calmette-Guérin for severe forms of tuberculosis (12.11%) by 24 months of age (Figure 1C).

The highest proportion of non-vaccination was observed for meningococcal vaccine (29.14%). This was observed in the municipalities of Fortaleza (29.82%) and Sobral (20.29%). The latter municipality also had a higher proportion of non-vaccination for other major vaccine-preventable diseases (poliomyelitis: 17.75%; measles, mumps, and rubella: 15.75% each).

Vaccinations against yellow fever showed very low uptake, with no doses administered (90.86%) in the municipalities of Fortaleza (96.69%) (Figure 1A) and Sobral (14.74%) (Figure 1B).

No covariates were excluded due to multicollinearity; VIF values for the final adjusted model are shown in Table 3. The Poisson model showed an association between non-vaccination against vaccine-preventable diseases and maternal ethnicity, specifically Caucasian (aPR 2.07, 95%CI 1.08-3.94; p = 0.028) and use of private vaccination services (aPR 1.72, 95%CI 1.46-2.04; p < 0.001) (Table 3).

**TABLE 3:** Prevalence ratio of non-vaccination in a cohort of live births in 2017 and 2018, by sociodemographic variables, use of vaccination services, and family, mother, and child characteristics, considering vaccination status up to 24 months of age (valid doses), living in the municipalities of Fortaleza and Sobral, Northeast Brazil.

Sociodemographic variables	Crude PR (non-vaccination) (95% CI)	p-value	Adjusted PR (non-vaccination) (95%CI)	p-value
**Use of private vaccination service**			**VIF: 1.02**	
Yes	1.76 (1.47-2.11)	<0.001	**1.72 (1.46**-**2.04)**	**<0.001**
No	1.00		1.00	
**Maternal characteristics**				
**Ethnicity**			**VIF: 1.02**	
Caucasian	2.44 (1.20-4.97)	0.014	**2.07 (1.08-3.94**)	**0.028**
Afro-Brazilian/Afro-descendant	1.00		1.00	
Mixed/Pardo Brazilians	1.82 (0.98-3.41)	0.059	Not retained	
Asian-descendant	0.65 (0.20-2.10)	0.473	Not retained	
Indigenous (Amerindians)	0.43 (0.03-5.37)	0.511	Not retained	

**PR:** prevalence ratio; **CI:** confidence interval; **BRL:** Brazilian Real; **VIF:** variance inflation factor. p-values are from survey-weighted Wald tests; for categorical variables, the reported p-value corresponds to the test by variable category. Categories not retained in the final adjusted model (p>0.05) are indicated as “not retained.”

## DISCUSSION

This study highlights that a high proportion of children were not vaccinated during the appropriate period (valid doses up to 24 months of age) against vaccine-preventable diseases, given the high occurrence of children with incomplete or no doses according to the vaccination schedules recommended by the national vaccination calendar of the PNI among live births in 2017 and 2018 in the municipalities of Sobral and Fortaleza, State of Ceará.

From a critical standpoint, the substantial proportion of children, either unvaccinated or receiving invalid doses administered outside the immunologically effective window, reflects a failure to ensure adequate immune protection and signals an epidemiological environment conducive to the resurgence of vaccine-preventable diseases^17^ ^-^ ^20^. Operationally, within Brazil’s SUS, such patterns reveal significant gaps in the continuity of care and immunization tracking, as evidenced by the declining adherence to multidose vaccine regimens^17^ ^,^ ^21^. This scenario fosters a misleading perception of coverage and child protection, often obscured by elevated dropout rates in completing the immunization schedules established by the PNI^6^. These specific multidose vaccines may have been abandoned owing to higher vaccination adherence in the first few months, which may be related to better access to routine checkups for children during that period^21^.

The non-vaccinated population surveyed was concentrated in both socioeconomic extremes in Fortaleza and Sobral. This pattern suggests that non-vaccination is shaped by multiple mechanisms: social vulnerability and access barriers in disadvantaged strata, and, in advantaged strata, factors such as vaccine hesitancy, fragmented follow-up, and the use of private services with incomplete integration into routine tracking. Our findings indicate that the unvaccinated child population exhibits high social inequality^1^ and heterogeneous barriers across the local health system and communities^17^ ^,^ ^18^ ^,^ ^21^ ^,^ ^22^.

During the study period, the yellow fever vaccine was recommended only in Ceará for people travelling to at-risk areas. This explains the low yellow fever vaccination coverage in the capital and other municipalities of the state, with the exception of Sobral and Cariri, where routine vaccination was initiated earlier (from 2020), and uptake may be higher because the vaccine is included in the national immunisation schedule^10^.

The risk of outbreaks of vaccine-preventable infectious diseases has reached its highest level in the Americas in the last three decades because of low vaccination coverage, a context that has been observed in the State of Ceará in recent years, leaving the child population susceptible to serious vaccine-preventable diseases^23^ as well as a decline in vaccination coverage that was aggravated during the coronavirus disease 2019 (COVID-19) pandemic^2^ ^,^ ^24^.

Although vaccination coverage increased in the State of Ceará in 2023^25^, failure to meet the targets proposed by the PNI has resulted in a scenario of risk and vulnerability, even in areas free of infectious agent circulation, particularly in the context of social inequality and limited access to health services^26^. The increase in international migration contributes to maintaining the international spread of vaccine-preventable diseases such as poliomyelitis^26^ ^,^ ^27^.

In this context, in 2021 and 2022, the endemic transmission of wild poliovirus in countries such as Afghanistan and Pakistan, the export of cases to other countries such as Malawi and Mozambique, and the reporting of cases of vaccine-derived poliovirus in the United States of America and Israel have raised awareness of the need to strengthen surveillance of vaccine-preventable diseases^28^.

The emergence of measles cases in Brazil from 2013 to 2015, including cases recorded in the State of Ceará, is concerning in light of the current situation, in which the minimum target recommended by the PNI has not been met despite recent increases in vaccination coverage^5^. This is compounded by Brazil's return to the United Nations Hunger Map amid food insecurity and the risk of increased severity and lethality of vaccine-preventable diseases such as measles^29^ ^,^ ^30^.

The high risk of severe cases of these diseases is evidenced by the fact that more than 7% (414/5,565) of Brazilian municipalities either have no available data on vaccination coverage (n=5) or report a vaccination rate of 0% among children (n=409)^19^. The critical nature of this scenario is further underscored by the fact that the occurrence of even a single case of certain diseases, such as poliomyelitis, would trigger urgent public health action and international notification requirements; poliovirus continues to be addressed under the WHO IHR Emergency Committee process^26^.

Given the state's migrant and tourist flows, understanding the populations most at risk of vaccine-preventable diseases and the availability of health information for management is essential for the timely implementation of effective control measures, such as timely surveillance and weekly reporting of notifiable diseases^18^. Identifying populations with incomplete or no vaccination, developing interventions to promote vaccination coverage, maintaining disease outbreak surveillance, and rescuing unvaccinated children are important public health interventions for greater immunological security^14^ ^,^ ^31^.

In the municipality of Sobral, although the population is fully covered by PHC, and therefore by vaccination services, more than half of the population remains economically disadvantaged, with an average monthly income of up to half the minimum wage per capita^32^, living in areas of lower socioeconomic strata, a context that may have influenced the restriction of access to vaccination for social, economic, and operational reasons and contributed to the increase in the unvaccinated population in this municipality. Additionally, gaps in access to rural areas, which are common in inland municipalities, may have contributed to the maintenance of this scenario^21^ ^,^ ^33^.

Conversely, our adjusted analysis (Table 3) indicates that non-vaccination was not confined to socially vulnerable groups; it was also associated with maternal self-identification as White and with the preferential use of private healthcare services. Even among populations of higher socioeconomic strata, numerous unvaccinated children persist, with low vaccination coverage among the White population^34^. This pattern may reflect a combination of behavioral, operational, and information system factors. First, reliance on private vaccination services can reduce routine linkages with primary care and public sector follow-ups, limiting opportunities for active search and timely catch-up of delayed doses. Second, operational barriers such as restricted opening hours, missed appointments, and scheduling difficulties may affect families across socioeconomic strata. Third, when doses are administered outside the public network, incomplete or inconsistent recordings in the SI-PNI, absence of standardized reporting routines, and variable adherence to multivaccination campaigns may compromise the accurate monitoring of immunization status and hinder targeted outreach in children with incomplete schedules^22^ ^,^ ^35^. Finally, vaccine hesitancy amplified by the rapid dissemination of misinformation, particularly during and after the COVID-19 pandemic, may also contribute to delayed or refused vaccinations, even in socioeconomically advantaged settings^36^.

This pandemic scenario is especially concerning given the significant role of vaccines in reducing the disease burden and transmission. Globally, vaccination is associated with an annual gain of approximately 3.84 million Quality-Adjusted Life Years and a projected net monetary benefit of 1-17 billion US dollars, highlighting its social impact and cost-effectiveness^37^. These challenges underscore the urgent need to integrate structured strategies in primary healthcare, including information, education, and communication actions, proactive outreach, continuous assessment, and systematic monitoring of immunization data through PNI information systems^38^ ^,^ ^39^. In other regions of Brazil (Southeast and Central-West), complete vaccination coverage was achieved in less than half of the children surveyed. The South and North regions had higher coverage but remained well below the target set by the Ministry of Health^40^ ^-^ ^43^. Furthermore, addressing the current scenario requires the revitalization of the PNI within the SUS; investment in epidemiological research; and the engagement and training of health workers, managers, educational institutions, mechanisms of social control, and civil society to restore and sustain high immunization coverage and ensure effective control of vaccine-preventable diseases among Brazilian children^2^ ^,^ ^33^ ^,^ ^39^ ^,^ ^44^ ^-^ ^46^.

Decisions to vaccinate children must be addressed and analyzed in light of legal regulations and the dimensions of individual, family, and parental responsibility^46^ ^,^ ^47^. Additionally, safe and effective vaccines against various diseases, such as the introduction of Inactivated Poliovirus Vaccine (IPV), have been produced to reduce the risk of susceptibility to vaccine-derived poliovirus^45^ ^,^ ^48^.

The limitations of this study include the limited access to households during data collection during the pandemic period, especially in the most advantaged socioeconomic strata; the use of population data from the demographic census conducted more than a decade ago (2010); the lack of standardized records of vaccine doses administered; and the limited quality of the photographs in the immunization records.

In conclusion, our findings highlight the high prevalence of non-vaccination among children born in 2017-2018 (vaccination status up to 24 months) against vaccine-preventable diseases in Fortaleza and Sobral, State of Ceará, Northeast Brazil, and identify the associated sociodemographic factors. In the adjusted model, maternal ethnicity (Caucasian category) and use of private vaccination services remained associated with non-vaccination. The risk of a resurgence of serious vaccine-preventable diseases has severe implications for public health policies and underscores the need for differentiated and more proactive strategies within the SUS implemented by managers and health professionals, with intensified vaccination according to local realities and microplanning.
